# Impact of Blood Metabolic Profile and Ingestive Behaviours Registered with Noseband Sensor on Methane Emission During Transition Period in Dairy Cows

**DOI:** 10.3390/life15050760

**Published:** 2025-05-09

**Authors:** Justina Krištolaitytė, Karina Džermeikaitė, Arūnas Rutkauskas, Greta Šertvytytė, Gabija Lembovičiūtė, Samanta Arlauskaitė, Akvilė Girdauskaitė, Violeta Juškienė, Remigijus Juška, Walter Baumgartner, Ramūnas Antanaitis

**Affiliations:** 1 Large Animal Clinic, Veterinary Academy, Lithuanian University of Health Sciences, Tilžės Str. 18, LT-47181 Kaunas, Lithuania; karina.dzermeikaite@lsmu.lt (K.D.); arunas.rutkauskas@lsmuni.lt (A.R.); greta.sertvytyte@lsmu.lt (G.Š.); gabija.lemboviciute@lsmu.lt (G.L.); samanta.arlauskaite@lsmu.lt (S.A.); akvile.girdauskaite@lsmu.lt (A.G.); ramunas.antanaitis@lsmu.lt (R.A.); 2 Department of Ecology, Animal Science Institute, Lithuanian University of Health Sciences, R. Zebenkos 12, 82317 Baisogala, Lithuania; violeta.juskiene@lsmu.lt (V.J.); remigijus.juska@lsmu.lt (R.J.); 3 Clinical Department for Farm Animals and Food System Science, Clinical Center for Ruminant and Camelid Medicine, University of Veterinary Medicine, Veterinaerplatz 1, A-1210 Vienna, Austria; walter.baumgartner@vetmeduni.ac.at

**Keywords:** methane emission, innovative technologies, dairy cows, blood samples

## Abstract

This study investigates the relationship between methane emissions and physiological, behavioural, and haematological parameters in dairy cows during the transition period. Methane emissions were monitored alongside variations in rumination, feeding behaviour, and blood markers three weeks before calving, on calving day, and three weeks post-calving. Cows were retrospectively classified into low, medium, and high rumination groups according to their average daily rumination duration to investigate the effects of behavioural influences. During the prepartum period, the methane concentration was moderately positively correlated with drinking time (r = 0.41, *p* < 0.01) and weakly negatively correlated with chews per minute (r = −0.358, *p* < 0.05). Significant negative correlations were noted with chloride (r = −0.42, *p* < 0.01) and glucose levels (r = −0.41, *p* < 0.01). Following calving, methane emissions showed a positive correlation with haematocrit (r = 0.41, *p* < 0.01) and a negative correlation with haemoglobin (r = −0.47, *p* < 0.01). A haematological analysis revealed a notable negative correlation with platelets during calving (r = −0.64, *p* < 0.05). Individual dry matter intake (DMI) was recorded for each period, showing a significant drop on calving day. This intake fluctuation coincided with a significant rise in methane yield on calving day (*p* < 0.001). In the low rumination time group, methane was moderately negatively correlated with rumination chews (r = −0.52, *p* < 0.05), while in the high rumination group, a moderate negative correlation was observed with drinking gulps (r = −0.42, *p* < 0.05), and a weak negative correlation was observed with bolus events (r = −0.37, *p* < 0.05). Despite behavioural variations, methane emissions showed no substantial differences among groups with low, medium, and high rumination times, suggesting a minimal direct influence on rumination duration. These findings emphasise the complex interactions between feed intake, metabolism, and methane emissions, underscoring the importance of integrating behavioural and physiological indicators to develop targeted strategies for enteric methane mitigation while providing baseline data from healthy cows that could guide future research on methane emissions in cows undergoing postpartum metabolic disorders.

## 1. Introduction

The emission of greenhouse gases (GHGs) by dairy farms has been recognised as a significant contributor to climate change, particularly in the context of initiatives like the European Green Deal, which underscores the need to mitigate methane emissions from agriculture to achieve climate neutrality by 2050 [1]. Nevertheless, cattle also play a vital role in sustainable food systems by converting fibrous, non-digestible plant material into high-quality protein for human consumption. Cattle and sheep farming are among the primary sources of agricultural GHG emissions, accounting for up to 18% of global agricultural GHG emissions, mainly due to enteric methane (CH_4_) production [2]. According to the United States Environmental Protection Agency, 37% of methane emissions result from anthropogenic livestock and agricultural practices [3]. A mature cow can emit up to 500 litres of methane daily, and the methane greenhouse effect is 25 times greater than that of CO_2_ [4,5]. Methane is produced in the rumen during the fermentation process by methanogenic archaea, which utilise CO_2_ and hydrogen (H_2_), methylamines, methanol, or acetate to generate CH_4_ [6]. While high-fibre diets can increase methane production due to shifts in ruminal fermentation, these diets are essential for ruminant health and function as they promote efficient digestion and nutrient absorption from forages that would otherwise be unusable in human diets [7]. The ability of ruminants to upcycle low-value plant material into high-quality protein contributes to food security and agricultural sustainability.

Developing strategies to reduce methane while maintaining the productivity and welfare of dairy cows is crucial for the sustainable development of the dairy industry [8]. Precision livestock farming (PLF) has emerged as an effective approach, using technologies such as sensors, data analytics, and automation to optimise farm management. These innovations not only enable targeted emission mitigation strategies, such as precision feeding and selective breeding for lower-emission animals, but also contribute to improving overall farm efficiency [9]. Despite this progress, a research gap remains in understanding the relationship between methane emissions and physiological, behavioural, and blood parameter changes, particularly during the transition period around calving, when cows undergo significant physiological adaptations [10]. Although many mitigation strategies focus on dietary interventions, the physiological status of the animal itself is an important determinant of methane emission. Thus, understanding how methane production is linked to health and behaviour during transition is essential. Integrating blood sample analysis—specifically the evaluation of metabolic and haematological parameters—with real-time behavioural monitoring technologies provides a deeper understanding of methane production mechanisms. These physiological indicators signify energy balance, metabolic stress, and immune status, all of which can affect feed intake patterns, rumen function, and, consequently, methane emissions. A comprehensive understanding of these connections may facilitate the development of targeted mitigation strategies designed to diminish the environmental impact of dairy farming while maintaining its essential role in global food systems.

Over time, various methods have been developed to reduce methane emissions from dairy cattle, such as feeding strategies, feed supplements, selective breeding, and manure management [2,11]. In addition to these practical approaches, recent studies have focused on identifying potential biomarkers related to methane emissions. For example, Mikula et al. [12] showed that extended ruminating periods are associated with reduced methane emissions and lower methane output per unit of milk in high-yielding dairy cows fed a maize silage-based partial mixed diet without pasture access. A recent study also found that rumination time was negatively correlated with methane production in first-lactation Holstein cows [13]. In contrast, not all studies have found consistent results. Zetouni et al. [8] did not observe any correlation between rumination time and CH_4_ emissions, likely due to insignificant variations in rumination time, though their study highlights a connection between CH_4_ and dry matter intake (DMI). Similarly, Watt et al. [7] found no difference between rumination time and CH_4_ production in grazing dairy cows. Increased feed digestibility has been shown to reduce methane production in dairy cows as more efficiently fermented feed results in lower enteric methane emissions [14]. Furthermore, cows selected for higher feed efficiency tend to produce less methane, which suggests that improved feed efficiency is linked to reduced methane emissions [15]. Reducing enteric methane production involves a combination of strategies, including methods and the mitigation and measurement of methane emissions. All methods aimed at reducing enteric methane should also consider the economic impact on farm profitability and the connections between enteric methane and other greenhouse gases.

During the transitional phase, dairy cows face a dysfunctional immune system and an increased inflammatory state due to the modulation of pathways related to metabolism, immune status, and the endocrine system [16]. Merdana et al. [17] conducted a study which aimed to determine the profile of erythrocytes, haemoglobin (Hb) levels, and haematocrit (Hct) values in Bali cattle during the periparturient period. The results of their study show that the changes in these blood parameters at different times of the transition period are related to calving, are not influenced by other factors, and return to standard limits.

In early lactation, the leukocyte proliferation response can vary due to metabolic and endocrine adaptations occurring during the transition period, a time when energy demands peak to support mammary gland function. The balance between energy intake and expenditure is critical, with negative energy balance primarily being driven by decreased feed intake during physiological stress and the increased energy demands of lactation and pregnancy [18]. Digestion during rumination plays a key role in ensuring an adequate energy supply. While methane production represents an inherent energy loss, it is a secondary factor compared to the major influences of feed intake and metabolic shifts [19]. Nonetheless, cows with higher methane-related energy losses may experience compounded effects on energy availability, potentially impacting immune function [20,21]. Notably, current research has predominantly focused on methane emissions through controlled feeding experiments or genetic traits, yet there is a lack of studies examining the physiological and behavioural parameters of healthy cows concerning methane production during the transition period. Based on the available literature, dairy cows undergo significant physiological and behavioural changes during the calving period, which are reflected in alterations in methane emissions, noseband sensor parameters (such as rumination time and feeding behaviour), and blood parameters (including haematological indices, mineral levels, and blood gases). These changes could serve as indicators for optimising health management and reducing the environmental footprint of dairy farming. Understanding methane emissions in clinically healthy cows during this critical transition phase provides a necessary physiological reference point for future studies evaluating how metabolic disorders may influence methane output through altered behaviour, feed intake, and blood profiles.

This study aims to comprehensively examine the physiological and behavioural changes in dairy cows during the calving period by assessing methane emissions and noseband sensor parameters, alongside blood parameters such as haematological indices, minerals, and blood gases, by analysing data across three key stages—prepartum, parturition, and postpartum—to explore their interrelationships and potential implications for cow health and environmental impact.

## 2. Materials and Methods

### 2.1. Housing Conditions of Study Animals

This study complied with the regulations outlined in the Lithuanian Law on Animal Welfare and Protection and was granted approval under the reference number PK012858. The research was carried out in Lithuania (55.636642, 23.716823) and took place from 24 November 2023 to 26 April 2024. A total of 13 clinically healthy dry Holstein cows were included in the study. The mean body weight of the cows was 650 ± 45 kg (SE). The group comprised multiparous cows, with an average parity of 2.8 ± 0.6. Cows were selected based on optimal health and production records to guarantee a homogeneous group without of metabolic disorders at the beginning of the study. Animals were housed tethered in stall barns and provided with a well-balanced total mixed ration (TMR) that was specifically designed to meet their physiological needs. Dry cow diets were designed to maintain a negative dietary cation–anion difference (DCAD) through the addition of anionic salts, with an average DCAD value of −100 mEq/kg dry matter, consistent with standard prepartum nutritional protocols, meant to enhance calcium mobilisation and avert hypocalcaemia [22,23]. Feeding times were scheduled daily at 08:00 and 18:00, with drinking water provided ad libitum. After calving, cows were transitioned to a lactation total mixed ration formulated to satisfy increased energy and nutrient requirements. The DCAD of the early lactation diet was modified to a positive value of approximately +250 mEq/kg dry matter, facilitating optimal rumen function and milk production during the postpartum period [23,24]. The detailed ingredient composition and chemical analysis of both diets are presented in Table 1 and Table 2, respectively. The average bunk space per cow was approximately 65 centimetres. Cows were milked twice daily at 07:00 and 17:00 in stall barns, starting after parturition, in accordance with the farm’s standard routine throughout the study period. During the previous lactation, the cows achieved an average energy-corrected milk yield of 12,500 kg per lactation (4.2% fat and 3.6% protein). The calculations were performed based on predetermined formulas.

### 2.2. Experimental Animals and Study Design

A total of 13 dry clinically healthy Holstein cows were enrolled in the study 40 days before calving. All cows were multiparous (mean parity 2.8 ± 0.6) and had calved easily in the previous lactation (1–3). The health status of these cows was checked daily by a local veterinarian throughout the study period, and only cows exhibiting no clinical signs of illness or metabolic disorder were included. The cows were observed during three specific physiological periods: the prepartum period (three weeks prior to calving), the calving period (calving day ±3 days), and the postpartum period (three weeks following calving). The selected time points were intended to encompass the critical metabolic and behavioural changes associated with parturition. All cows functioned as their own control in a repeated-measures observational design. No experimental interventions were carried out. RumiWatch noseband sensors (RWS) (RumiWatch System, Itin+Hoch GmbH, Liestal, Switzerland) were applied on each cow roughly 30 days prior to calving. The sensors consistently documented feeding, rumination, and activity parameters during the study. Data were analysed by comparing physiological and behavioural measurements within each cow across the three intervals.

### 2.3. Measurement of Variables

#### 2.3.1. Methane Emission Measurement

The laser methane detector (LMD), an innovative, non-invasive technology that provides accurate, real-time data on methane production, was validated as a reliable tool for measuring enteric methane emissions in dairy cattle. Studies, such as that by Sorg et al. [25], demonstrated a high correlation (R^2^ ≈ 0.9) between LMD measurements and those obtained from respiratory chambers, confirming the consistency of LMD devices and their ability to provide precise data without disrupting normal cow behaviours.

The portable LMD HESAI HS4000 (Hesai, Building L2-B, Hongqiao World Centre, Shanghai) utilises infrared absorption spectroscopy to measure methane concentrations. The integrated concentration of CH_4_ between the LMD and the target is determined by detecting diffusely reflected laser light [26]. The measured value is expressed as the CH_4_ column density (ppm), which represents the integrated CH_4_ concentrations along the laser path. This value is calculated as the product of the average CH_4_ concentration (ppm) and the path length (m) [27]. LMD can measure CH_4_ concentrations ranging from 0.5 to 50,000 parts per million by volume (ppm), equivalent to a range of up to 5% by volume. Furthermore, the LMD can be efficiently used at distances between 0.5 and 30 m. Measurements are displayed in real time on the LMD’s screen, and an audio-visual alert is triggered if a predefined threshold is exceeded. Methane expelled from the rumen through eructation is first inhaled into the lungs and then exhaled during each respiratory cycle. These emissions were measured by targeting the nostril area, with all measurements conducted by a single operator to ensure consistency throughout the study. The veterinarian manually operated the LMD, tracking each animal’s head movements to measure exhaled CH_4_ concentrations. Measurements were taken twice per day, with each session lasting at least three minutes, resulting in a total of approximately 360–720 CH_4_ values per animal per day, captured at a frequency with measurement intervals of 0.5 to 1 s, thus recording one or two CH_4_ values per second.

All measurements were taken at a consistent time, approximately two hours after the morning feeding, to control for diurnal variations in methane emissions. On the day of parturition, measurements were taken only after calving, once the cow had stabilised and consumed feed [28]. Before the study, the LMD was calibrated using the manufacturer’s standard calibration files, which encompass laser-specific elevation and azimuth parameters. No supplementary third-party calibration was executed; however, field-level consistency was preserved by conducting measurements in low-wind conditions, avoiding reflective surfaces, and standardising the operator’s distance and angle to the target area [29]. These procedures conform to established standards for minimising environmental interference and ensuring accurate methane measurements in live animal environments.

#### 2.3.2. Blood Sample Analysis

Blood samples were collected from the coccygeal vein once per week starting three weeks before the expected calving date and continuing until three weeks postpartum. Additionally, a sample was taken during the calving period, defined as the day of calving ±3 days, to represent the periparturient phase. All blood collection procedures were carried out after the morning milking and before the morning feeding. Samples for morphological analysis were taken in 10 mL EDTA tubes (Vacuette^®^, Greiner BioOne GmbH, Kremsmünster, Austria) and transported to the Laboratory of Clinical Tests at the Large Animal Clinic, Lithuanian University of Health Sciences Veterinary Academy. Blood samples for measuring blood gases and the other metabolic markers were collected in vacuum tubes with lithium heparin (BD Vacutainer^®^, Becton, Dickinson and Company, Plymouth, Devon, UK) and kept in an ice bath until all samples were taken. Blood gases (hydrogen potential (pH), partial pressure of carbon dioxide (pCO_2_), partial pressure of oxygen (pO_2_), concentration of bicarbonate (cHCO_3_^−^), base excess of extracellular fluid (BE (ecf)), and oxygen saturation of haemoglobin (cSO2)) along with biochemical and haematological parameters (sodium (Na), potassium (K), calcium (Ca), chlorides (Cl), total carbon dioxide (tCO_2_), haematocrit (Hct), haemoglobin (Hgb), base excess in blood (BE (b)), glucose (Glu), lactate (Lac), blood urea nitrogen (BUN), urea nitrogen (Urea), and creatinine (Crea)) were analysed using an Epoc ^®^ blood gas analyser (EPOC, Ottawa, ON, Canada).

#### 2.3.3. Behavioural Monitoring with RumiWatch Sensors

RumiWatch noseband sensors (RWSs) (ITIN+HOCH GmbH, Liestal, Switzerland) were attached to the cows’ heads to collect detailed behavioural data, including rumination time, drinking time, and overall activity levels. These sensors have been validated in previous studies for monitoring diverse physiological parameters [30,31]. The use of RWSs represents significant progress in monitoring bovine behaviour, allowing for meticulous and uninterrupted data gathering. The RWS consists of a pressure tube filled with liquid and a noseband halter that is equipped with a pressure detector. The system algorithms classify behaviours by identifying unique clusters of pressure peaks generated by movements of the jaw and categorise them based on specific behavioural characteristics. The RWS systems documented rumination, eating, and locomotion behaviours, enabling the computation of different parameters. The measured variables consisted of rumination time (duration of rechewing regurgitated cud until re-swallowing, including breaks of up to 5 s), eating time (duration of chewing food, including breaks of up to 5 s), and drinking time (duration of drinking, including delays of up to 5 s between gulps). The additional parameters that were recorded include rumination chews (chewing of molars during rumination), eating chews (total number of bites and mastication chews during eating), drinking gulps (total number of gulps taken while drinking), bolus (total amount of gulps during drinking), activity (duration of walking bouts presented as minutes within a given period), up time (time spent feeding with the head positioned upwards), down time (time spent feeding with the head positioned downwards), and activity change (number of transitions between activities such as other activity, ruminating, eating, and drinking). The primary algorithms of the RWC software (version 0.7.3.2) facilitate the accurate classification of behavioural 10 Hz pressure data components across various time summaries. The algorithms identify distinct pressure peak clusters resulting from jaw movements and categorise them according to their behavioural characteristics [32,33].

#### 2.3.4. Individual Dry Matter Intake (DMI) Calculation

To assess the relationship between individual DMI and methane emissions, daily feed intake measurements were conducted for each cow. Feed intake was recorded by weighing the total feed offered to each cow before feeding and then collecting and weighing the feed refusals after a 24 h feeding period. The difference between these values provided the actual feed consumed per cow per day. The individual DMI was calculated using the following formula:

$${DMI}_{i}=\frac{{Feed\;Intake}_{i}\times DM\;\%\;of\;Diet}{100}\;$$

where

DMI_i_—the estimated daily dry matter intake of an individual cow (kg);
Feed Intake_i_—the total feed consumed per cow (kg/day);
DM % of Diet—the dry matter percentage of the total mixed ration (TMR).

### 2.4. Duration of Experimental Observation

The cows were monitored and sampled at three critical stages of the peripartum period: 3 weeks before calving (prepartum), during the week of calving (parturition; calving day ± 3 days), and 3 weeks after calving (postpartum). These stages were selected based on standard transition cow research protocols to capture the significant physiological and metabolic changes occurring during this period. All cows were equipped with RWS approximately 30 days before calving, allowing for a seven-day adaptation period. Rumination and eating parameters were continuously recorded by RWSs starting from 30 days before calving to 30 days after calving. Weekly blood samples were collected on a fixed schedule, while methane emissions were measured daily throughout the entire seven-week study period.

To investigate potential associations between rumination behaviour and enteric methane production, cows were retrospectively categorised into three rumination classifications based on their average daily rumination duration: low rumination (<404 min/day), medium rumination (404–500 min/day), and high rumination (>500 min/day). The categories were established based on previously determined average rumination durations in high-yielding dairy cows, which generally range from 440 to 460 min daily [8,12]. A comparative analysis of methane emissions among these rumination categories was performed to assess potential correlations between rumination behaviour and enteric methane production.

### 2.5. Statistical Analysis

All statistical analyses were conducted using version 25.0 of IBM SPSS Statistics for Windows (IBM Corp., Armonk, NY, USA). To assess the normality of the data distribution, the Shapiro–Wilk test was utilised. Data were presented as the mean plus/minus the standard error of the mean (M ± S.E.M.). One-way ANOVA and a general linear model with repeated-measures tests were applied for repeated measurements, encompassing time intervals with an identical RumiWatch, blood parameters, and methane emission indicators during different experimental periods. The Pearson’s correlation coefficient was calculated to identify the linear relationship between variables. A *p*-value of less than 0.05 was considered statistically significant (*p* < 0.05). A Principal Component Analysis (PCA) was performed to ascertain the correlation among different variables.

## 3. Results

### 3.1. Effects of Prepartum and Postpartum Periods on Methane Emissions in Dairy Cattle

**Figure 1:**
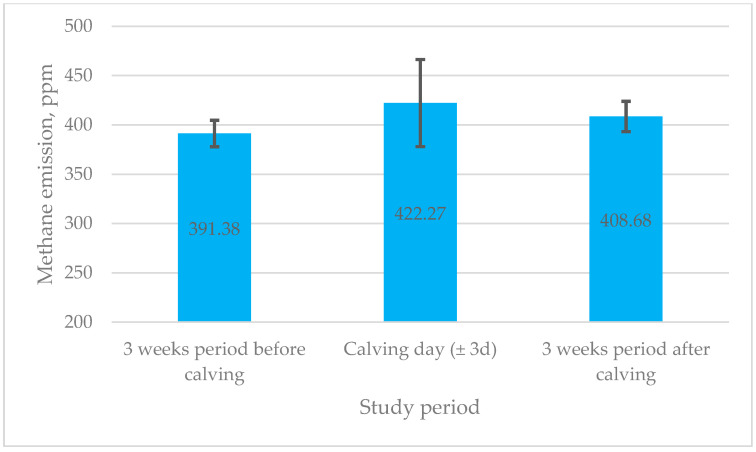
Mean methane emissions across different transition periods in dairy cows. ppm—parts per million; d—day.

An examination of research data revealed that the cows, on calving day, had a tendency to produce higher methane emissions (Figure 1).

Nevertheless, there were no significant changes between different transition periods, i.e., emissions of methane increased by only 30.89 ppm on calving day compared to 3 weeks before calving and increased by 13.60 ppm 3 weeks after calving (*p* > 0.05).

The study’s data indicate significant differences (*p* < 0.05) in the rumination time throughout the three observed times around calving. Three weeks before calving, the cows showed an average rumination time of around 435.71 min. On the day of calving (±3 days), the rumination time decreased to an average of 403.94 min, indicating a drop of about 7.3% from the pre-calving period.

During the three weeks post-calving, the rumination duration significantly rose to an average of 504.55 min per 24 h. This increase corresponds to around 16% relative to the pre-calving levels and approximately 24.9% in comparison to the calving period (*p* < 0.05).

The duration of eating diminished by 14.28% on calving day relative to the three weeks preceding calving, yet it escalated by 35.08% in the three weeks after calving (*p* < 0.01). Rumination chews decreased by 8.35% relative to the three weeks preceding calving. A 20.79% decrease was observed compared to the three weeks post-calving (*p* < 0.01). The rate of chews per minute exhibited a 7.29% reduction relative to the three weeks preceding calving. It was 12.5% lower compared to the three weeks post-calving (*p* < 0.01). The number of chews per bolus rose by 14.25% during the calving day relative to the pre-calving phase but subsequently decreased by 17.01% post-calving (*p* < 0.01). The number of boluses decreased by 7.8% on calving day relative to pre-calving but rose by 12.48% post-calving compared to pre-calving (*p* < 0.05) (Table 3).

### 3.2. Correlations Between Methane Emission and Activity Variables of Prepartum and Postpartum Periods

The methane amount exhibited a moderate positive correlation with drinking time (r = 0.41, *p* < 0.01) and a weak negative correlation with chews per minute (r = −0.358, *p* < 0.05) only during the three weeks before calving in cattle (Table 4).

### 3.3. Correlations Between Methane Emission and Blood Parameters in Prepartum and Postpartum Periods

The examination of methane emissions in dairy cows identified many significant relationships with physiological indicators throughout the transition phase. Three weeks before calving, methane emissions exhibited a moderate positive correlation with base excess in extracellular fluid (BE ecf) (r = 0.37, *p* < 0.05). A significant negative connection was identified with chloride (Cl) levels (r = −0.42, *p* < 0.01) and glucose (Glu) (r = −0.41, *p* < 0.01). Calcium (Ca) had a slight negative connection with methane emissions (r = −0.33, *p* < 0.05). The examination of methane emissions in dairy cows identified many significant relationships with physiological indicators throughout the transition phase. Three weeks before calving, methane emissions exhibited a moderate positive connection with base excess in extracellular fluid (BE ecf) (r = 0.37, *p* < 0.05). A significant negative connection was identified with (chloride) Cl levels (r = −0.42, *p* < 0.01) and Glu (r = −0.41, *p* < 0.01).

Three weeks post-calving, methane emissions exhibited a positive correlation with haematocrit (Hct, r = 0.41, *p* < 0.01) and a negative correlation with haemoglobin concentration (Hgb, r = −0.47, *p* < 0.01) and blood base excess (BE (b), r = −0.36, *p* < 0.05) (Table 5).

A significant correlation between methane and haematological blood parameters was also noted. Three weeks before calving, methane levels exhibited a strong positive connection with monocyte counts (r = 0.50, *p* < 0.01). A significant negative correlation was seen between methane emissions and platelet counts during calving (r = −0.64, *p* < 0.05). This negative correlation with platelets persisted into the postpartum period, with a moderate correlation being observed three weeks after calving (r = −0.41, *p* < 0.01) (Table 6).

### 3.4. Impact of Dairy Cows’ Ingestive Behaviours Registered with RumiWatch Noseband Sensor on Methane Emissions

**Figure 2:**
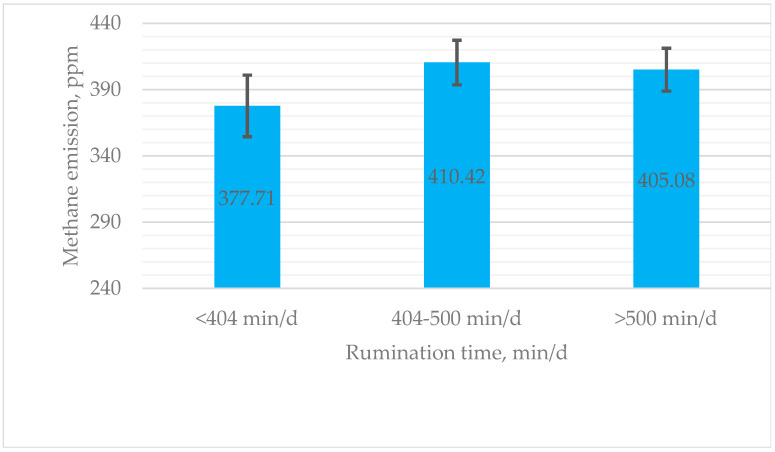
Estimated means for methane production (ppm) of cows with low (<404 min/d), medium (404–500 min/d), and high (>500 min/d) rumination. ppm—parts per million; min—minute.

An analysis revealed that cows who ruminate longer have a tendency to produce higher methane emissions. Cows exhibiting high rumination emitted, on average, 7.3% more methane than those with low rumination, while cows with medium rumination emitted 8.7% more methane than their low-rumination counterparts (Figure 2).

Although emissions of methane increased by 27.36 ppm in the high rumination time group compared to the low rumination time group, there were no significant changes between different rumination time groups (*p* > 0.05). The difference in methane emission results between the middle and high groups was 5.34 ppm (Figure 2).

### 3.5. Correlations Between Methane Emissions and Activity Variables in Different Rumination Times

A data analysis revealed that in the low rumination time group, methane was negatively correlated with rumination chewing time (r = −0.52, *p* < 0.05). Additionally, there was a weak positive correlation between methane and drinking gulps (r = −0.42, *p* < 0.05) and bolus (r = −0.37, *p* < 0.05) in the high rumination time group (Table 7).

**Figure 3:**
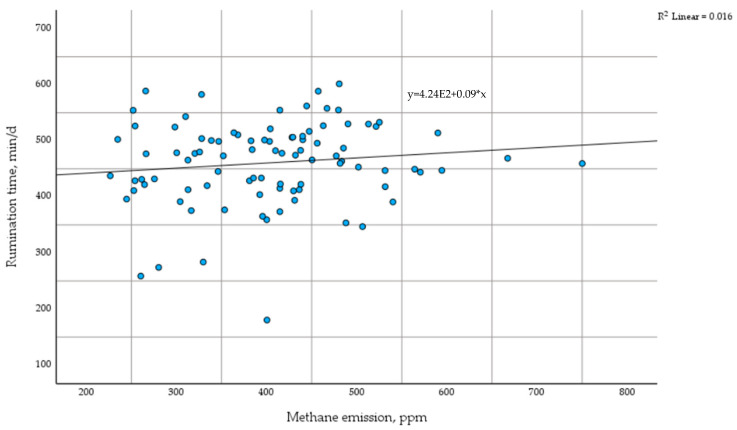
Differences in methane emissions based on time of rumination. min—minute; d—day; ppm—parts per million.

The scatter plot shows the relationship between daily rumination time and methane emissions in dairy cows during the transition period. The trendline indicates a weak positive correlation (R^2^ = 0.016) (Figure 3). 

The DBSCAN (Density-Based Spatial Clustering of Applications with Noise) algorithm was used to conduct a clustering analysis on methane and rumination time. No correlation was identified that would facilitate the categorisation of cows into separate groups. The correlation was 0.13, signifying an exceedingly weak relationship.

Subsequently, a Principal Component Analysis (PCA) was performed to ascertain the correlation among different variables. The PCA utilised six components, accounting for 79.97% of the data (Table 8).

A PCA with Oblimin rotation revealed that Component 5 demonstrates unique associations between different cow behavioural metrics and methane emissions, highlighting factors that may affect or be correlated with methane output. Methane has a negative loading of −0.62. The negative connection indicates that behaviours linked to Component 5 could work as markers of reduced methane emissions. Significant factors in this component are other chew (0.65) and activity (0.77), both exhibiting positive loadings. This indicates that heightened overall activity and chewing behaviour (excluding primary rumination) are linked to this component and negatively connected with methane emissions (Table 9).

### 3.6. Impact of Dry Matter Intake on Dairy Cows’ Methane Emissions

Following the analysis of behavioural patterns and their relationship with methane emissions, individual dry matter intake was examined to assess variations across the transition period and its potential impact on methane yield.

Methane yield (ppmCH_4_/kg DMI) was significantly higher on calving day (*p* < 0.001) compared to prepartum and postpartum periods, which did not differ significantly from each other (*p* > 0.05) (Table 10). These findings suggest that feed utilisation efficiency was the lowest on calving day, likely due to rumen fermentation disruptions, lower intake and physiological stress, metabolic adjustments, and reduced rumen capacity during parturition. Intake increased postpartum, aligning with the cows’ higher energy requirements for lactation and recovery from the transition phase.

Pearson’s correlation analysis revealed a moderate negative correlation between DMI and methane yield (r = −0.42, *p* < 0.05), suggesting that higher feed intake was associated with lower methane production per unit of dry matter consumed (Table 11).

## 4. Discussion

Numerous animal studies have been undertaken in recent decades to examine the behavioural alterations that occur in cows prior to calving [34,35,36]. Consequently, maintenance behaviours, including locomotor and postural activities (standing, lying down, and walking), alongside self-grooming and ingestive actions (eating, drinking, and ruminating), have been investigated [37,38]. This study aimed to explore the relationships between feeding and activity characteristics recorded by the RumiWatch noseband sensor—including the rumination time, drinking time, eating time, rumination chews, eating chews, drinking gulps, bolus count, chews per minute, and chews per bolus and activity—and methane emissions in dairy cows before, during, and after calving. While the RumiWatch system has been validated in previous research [31,32,39,40], the present study utilised this technology as a tool to capture detailed behavioural patterns. Importantly, this study is among the first to integrate RumiWatch data with haematological and metabolic blood parameters and daily methane measurements throughout the transition period. The results demonstrate that while there were no significant variations between transition periods, methane emissions peaked on calving day (mean: 422 ppm) and were lower three weeks before (391 ppm) and three weeks after calving (408 ppm) (*p* > 0.05).

A decreased rumination time is a characteristic change observed on the day of calving. Parturition is associated with an increase in lying events and a reduction in rumination chewing. The decline in the number of chews on calving day may be attributed to the pain and discomfort associated with the calving process itself [41]. Antanaitis et al. [31] reported similar findings, noting minimal variations in mean chewing activity, with a 39.37% increase observed on the ninth day postpartum compared to one day postpartum (*p* < 0.05). Similarly, Soriani et al. [42] demonstrated a significant reduction in the rumination time on the day of calving, reaching its lowest point (262–278 min/d), highlighting the physiological and behavioural stress cows experience during parturition. This stress may compromise rumen function and feeding behaviour. The observed decrease in rumination on the day of calving is likely due to pain and stress, which disrupt normal feeding and chewing patterns, reduce saliva production, and alter rumen fermentation, potentially influencing methane emissions.

Knapp et al. [43] indicated that diets with higher energy content or improved digestibility enhance net energy intake. When this energy is directed toward milk production, methane emissions per unit of energy-corrected milk (ECM) output tend to decrease. This study uniquely tracked daily dry matter intake and methane output in individual cows, demonstrating how physiological disruption at calving significantly impairs feed efficiency and elevates methane yield. The significant reduction in DMI on calving day (6.12 ± 1.83 kg DM/day) coincided with the highest methane yield (69.01 ± 5.6 ppmCH_4_/kg DMI, *p* < 0.001), suggesting a period of reduced feed efficiency and altered rumen fermentation. While absolute methane emissions peaked on calving day (422.27 ± 44.11 ppm), the elevated methane yield per unit of intake indicates less efficient fermentation, likely due to metabolic stress and lower rumen microbial activity [18].

The negative correlation between DMI and methane yield (r = −0.42, *p* < 0.05) further supports that cows with lower feed intake had higher methane yield, possibly due to reduced propionate production or altered hydrogen utilisation pathways. Previous studies have demonstrated that low DMI during the transition period alters volatile fatty acid (VFA) production, leading to a greater acetate-to-propionate ratio, which is linked to increased methane production per unit of feed intake [44,45]. Propionate acts as a hydrogen sink in the rumen, reducing the availability of hydrogen for methanogenesis; thus, decreased DMI may shift fermentation patterns toward greater acetate production, favouring methanogenesis [46]. These findings support the hypothesis that the metabolic and physiological stress experienced during calving can exacerbate methane emissions per unit of intake, a key metric in environmental efficiency.

Monitoring and optimising specific blood parameters in cattle may enhance overall health, improve efficiency by reducing methane emissions, and contribute to better growth performance in progeny, thereby increasing profitability [47]. Both macro- and microminerals play crucial role in optimising production performance by meeting fundamental physiological requirements. Their presence in the circulation is essential for various physiological functions, maintaining health, supporting growth and reproduction, and ensuring the proper function of the immune and endocrine systems [48]. Calcium ions (Ca^2+^) are particularly vital for muscular contraction as they facilitate the interaction between actin and myosin, leading to muscle contractions. In smooth muscles, such as those in the abomasum of cows, fluctuations in calcium concentrations influence both the intensity and frequency of contractions [49]. Furthermore, Hansen et al. [50] reported that the infusion of Na2EDTA, which induces subclinical hypocalcaemia (SCH; 0.9 mM iCa), led to reductions in chewing activity and dry matter intake (DMI) in nonlactating dairy cows. Given the role of calcium in regulating feed consumption and digestive efficiency, the blood calcium concentration may indirectly influence methane emissions. The present findings indicate a moderate negative association between blood calcium and methane emissions (r = −0.33, *p* < 0.05), suggesting that higher calcium concentrations may be associated with reduced methane production in dairy cows. This observation may be particularly relevant when making a comparison with cows with subclinical hypocalcaemia, which will be explored in future studies.

In ruminants, the liver plays a crucial role in maintaining energy balance by synthesising glucose from propionic acid absorbed from the rumen through the gluconeogenesis pathway. Additionally, it regulates lipid metabolism via fat oxidation and synthesis, both of which are essential for physiological functions [51,52]. Approximately 80–85% of ruminal propionate is transported to the liver via the portal vein, where it serves as the primary substrate for gluconeogenesis, while the remaining 15–20% circulates to other organs [44]. Kim et al. [45] reported that blood glucose concentrations did not differ between high- and low-methane-emitting cattle, suggesting that high-emission cows may sustain glucose production by adjusting gluconeogenesis to compensate for lower ruminal propionate availability and increased energy loss due to methane production. In contrast, the present findings do not support this, as the cows exhibited a moderate negative correlation between blood glucose levels and methane emissions three weeks before calving (r = −0.411, *p* < 0.01). The observed decrease in blood glucose levels during winter conditions may be attributed to reduced energy intake, potentially resulting from increased thermoregulatory demands and a downregulation of gluconeogenesis as an adaptive endocrine response to cold stress [53]. The observed inverse correlation between glucose and methane substantiates the notion that diminished energy levels during the prepartum phase could worsen methane-associated energy deficits, underscoring the necessity of metabolic monitoring. Meese et al. [20] suggested that in cows with low immune response, methane emissions per unit of body weight (BW) or energy-corrected milk (ECM) were reduced, potentially due to a reallocation of energy utilisation. A decreased energy allocation to immune activation resulted in greater energy availability for rumen fermentation and propionate synthesis, which in turn reduced methane production. In the present study, a significant positive correlation was consistently observed between methane emissions and monocyte counts (r = 0.50, *p* < 0.01) prior to calving, further supporting the notion that increased immune activity, as indicated by elevated monocyte levels, may contribute to higher methane emissions by influencing energy distribution and microbial fermentation processes.

Moderate blood loss during and after parturition, along with concurrent haemoconcentration, may also affect methane emissions [10]. A recent study indicated that, three weeks after calving, methane emissions exhibited a positive correlation with haematocrit (r = 0.41, *p* < 0.01) and a negative correlation with haemoglobin concentration (r = −0.47, *p* < 0.01). A notable negative correlation was identified during calving and at three weeks postpartum between methane emissions and platelet counts (r = −0.64, *p* < 0.05 and r = −0.41, *p* < 0.01). While platelets are primarily linked to blood clotting, they also indicate inflammatory responses. Haemoglobin and haematocrit reflect oxygen transport capacity, which is linked to metabolic rate. The observed increase in haematocrit after calving could be partially influenced by a transient dehydration effect due to the shift to lactation, as cows experience increased water demand and fluid losses through milk production [54]. This phase is also characterised by a substantial increase in feed intake, with prepartum cows consuming 13.92 ± 2.87 kg DM/day, calving day intake dropping significantly to 6.12 ± 1.83 kg DM/day, and postpartum intake rising to 21.34 ± 3.52 kg DM/day. Given that methane emissions are directly linked to feed intake and energy metabolism, changes in haematocrit may reflect both hydration status and metabolic shifts, which could influence methane production [55]. Since methane emissions are closely linked to feed intake and energy metabolism, fluctuations in haematocrit levels may not only reflect hydration status but also underlying metabolic adaptations affecting methane production [52]. These findings emphasise the need for nutritional strategies that support stable intake during the transition period, potentially mitigating methane inefficiencies while ensuring optimal metabolic adaptation postpartum. Strategies such as gradual dietary transitions, maintaining fibre intake, and providing sufficient energy-dense feeds without excessive starch may help optimise rumen fermentation dynamics and reduce methane yield per unit of feed intake [43].

It was hypothesised that extended rumination duration would correlate with reduced daily methane emissions in high-yielding dairy cows during the transition period. Cows across all groups ruminated for approximately 461 min per day, which is consistent with the findings of Mikula et al. [12], who reported a mean rumination time of 458 min per day, and Zetouni et al. [8], who documented an average rumination time of 443 min per day during the lactation of Danish Holstein cows.

Methane emissions varied among rumination time categories, with cows in the medium rumination group (404–500 min/day) showing the highest methane emissions. Cows exhibiting the longest rumination times (>500 min/day) produced marginally lower methane emissions, whereas the lowest methane emissions were observed in the low-rumination group (<404 min/day). The results contradict the findings of Mikula et al. [12], who reported that an increased rumination time had a positive influence, causing a reduction in methane production. In the study conducted by Castaneda et al. [56], high ruminating cows (404 ± 6.04) emitted significantly less methane (*p* = 0.003) than low ruminating cows (430 ± 6.27). Similarly, López-Paredes et al. [57] observed a negative genetic association between methane emissions and rumination duration. These findings differ from those of Zatouni et al. [8], who found no correlation between rumination duration and methane emissions in high-yielding dairy cows.

Phenotypes, methane emissions, and rumination activity are influenced by various quantifiable factors, leading to a wide range of results across different studies. Rumination increases the surface area of feed particles, facilitating microbial access, while also promoting saliva production to buffer the rumen and maintain a homeostatic environment for microbes [58]. More thorough chewing of fibrous material enhances the production of volatile fatty acids, which serve as essential energy sources for the animal [59]. This increased fermentation, though beneficial for energy yield, can also lead to higher methane production as methanogens in the rumen use hydrogen released during fermentation to produce methane [7]. A reduction in neutral detergent fibre from forage, combined with an increase in concentrate consumption, may lead to a decline in the rumen pH value. This shift could result in increased propionate levels and lower acetate and butyrate levels, as well as a reduction in hydrogen equivalents that would typically be converted to methane and serve as inhibitors in methanogenesis [12,60]. Watt et al. [7] demonstrated that cows with high rumination during grazing exhibit elevated methane emissions, corroborating the findings of the present study. While it was initially assumed that cows with high rumination during grazing would be heavier than those with low rumination, the current investigation found that high ruminating cows had a lower body weight.

Cameron et al. [61] identified a direct correlation between chewing rate and methane production, particularly within the range of 68–120 chews per minute, with higher chewing rates corresponding to increased methane emissions.

An analysis of the data revealed a somewhat negative correlation between methane emissions and rumination chew times in the low rumination group (r = −0.52, *p* < 0.05). Additionally, in the high rumination group, there was a weak positive correlation between methane emissions and drinking gulps (r = −0.42, *p* < 0.05) as well as bolus (r = −0.37, *p* < 0.05). These findings suggest that in cows with reduced rumination, an increase in rumination chew duration may be associated with lower methane production, indicating a possible inverse relationship. Additionally, during the prepartum period (three weeks prior to calving), the methane concentration demonstrated a moderate positive correlation with drinking duration (r = 0.41, *p* < 0.01) and a weak negative correlation with chews per minute (r = −0.36, *p* < 0.05). The absence of a definitive correlation between rumination duration and methane in this study indicates that alternative behaviours—such as chewing efficiency, drinking habits, and metabolic indicators—might exert a more direct influence on enteric methane production.

Balancing rumination and water intake is essential for maintaining efficient fermentation and reducing methane emissions. Water intake is known to temporarily, yet significantly, lower rumen or reticular temperatures, an effect associated with a decrease in microbial activity [62]. In sheep, water at 0 °C inhibited microbial activity, as evidenced by increased rumen pH and reduced levels of volatile fatty acids and ammonia-N [63]. Cold weather increases cattle’s energy requirements for thermoregulation, which can influence feed intake and digestion processes. The consumption of heated water has been shown to reduce the duration (min/day) during which ruminal pH falls below 5.8 or 5.5, as well as the time when rumen temperature drops below 37 or 39 °C (*p* < 0.001) [64]. The strategic use of heated drinking water may therefore serve as a viable approach to enhancing ruminal stability, microbiota composition, and fermentation efficiency in cattle. These findings suggest that monitoring cow behaviour using advanced sensors not only supports welfare assessment but may also provide real-time insights into methane emission trends and metabolic status.

Given the statistically negligible direct correlation between methane emissions and bovine behaviour, a PCA was conducted to identify deeper associations among the examined parameters. Component 5 exhibited significant loadings for “Methane Emission” (loading of −0.57) and “Activity” (loading of 0.54). The negative association between these variables suggests that as activity increases, methane production tends to decrease, or vice versa. This component may reflect the balance between metabolic energy allocated to movement versus digestion, with potential implications for methane production. Higher methane emissions could be associated with reduced physical activity, possibly due to a metabolic shift prioritising digestion over mobility. The findings suggest that activity and chewing are the primary behaviours affecting methane emissions, whereas variables like rumination time and rumination chews did not significantly contribute to Component 5. This indicates that methane emissions are more closely linked to the cow’s physical activity and mastication patterns than to rumination behaviour.

## 5. Conclusions

This study elucidates intricate relationships between methane emissions and physiological, behavioural, and haematological parameters in dairy cows during the transition period. Methane production exhibited significant correlations with key blood markers and activity parameters, highlighting the complex impact of metabolic and physiological changes on greenhouse gas emissions. Prior to calving, methane emissions showed a moderate correlation with base excess in extracellular fluid (r = 0.37) and a negative association with chloride (r = −0.42), glucose (r = −0.41), and calcium ((r = −0.33) concentrations, suggesting that metabolic alterations influence rumen fermentation dynamics. During and post-calving, methane emissions were associated with haematological changes, exhibiting an inverse correlation with haemoglobin (r = −0.47) and platelet levels (r = −0.64), which may reflect stress and metabolic adaptations in the periparturient phase.

Despite variations in the rumination time and mastication activity, methane emissions did not differ significantly among high, medium, and low rumination groups, indicating that factors beyond chewing behaviours influence emissions. Weak correlations with chews (r = −0.52), drinking gulps (r = −0.42), and bolus counts (r = −0.37) suggest that feeding and activity patterns contribute to methane variability. Given that feed intake plays a fundamental role in methane production, data on dry matter intake were incorporated to provide additional context for the observed patterns. The recorded DMI values showed a substantial reduction on calving day, decreasing by approximately 56% compared to the prepartum levels, followed by a 53% increase postpartum. This decline in intake during calving coincided with a 145% rise in methane yield, suggesting reduced feed efficiency and altered rumen fermentation during this period. In contrast, the methane yield decreased by 32% postpartum, aligning with the restoration of normal intake patterns.

This study offers a thorough baseline dataset on methane dynamics in healthy cows during the transition period by incorporating real-time sensor technologies and blood biomarkers. This reference framework may facilitate future research evaluating the impact of postpartum metabolic disorders—such as ketosis or hypocalcaemia—on methane emissions, thereby assisting in the creation of diagnostic or mitigation tools. Further research integrating nutritional, physiological, and environmental factors is essential for developing sustainable mitigation strategies that support both productivity and animal welfare.

## Figures and Tables

**Table 1:** Ingredient composition (% of DM) of total mixed rations for dairy cows.

Parameters	Dry Cows	Fresh Dairy Cows
Corn silage	—	25.0
Alfalfa grass hay	—	5.0
Grass silage *	44.1	20.0
Wheat straw	41.3	—
Maize silage	6.6	—
Rapeseed meal	6.6	—
Sugar beet pulp silage	—	15.0
Grain concentrate mash	—	30.0
Mineral and vitamin mix	1.4	5.0

* Grass silage was made of perennial ryegrass (*Lolium perenne*), timothy (*Phleum pratense*), and meadow fescue (*Festuca pratensis*).

**Table 2:** Chemical composition of total mixed rations for dairy cows.

Parameters	Units	Dry Cows	Fresh Dairy Cows
Dry matter	%	55.9	48.8
Net energy lactation	Mcal/kg	1.02	1.6
Crude protein (%DM)	%	8.9	15.8
Non fibre carbohydrates (%DM)	%	16.0	38.7
Neutral detergent fibre (%DM)	%	63.1	28.2
Acid detergent fibre (%DM)	%	38.6	19.8
Ether extract (EE) (%DM)	%	2.6	4.5
Acid detergent lignin (ADL) (%DM)	%	3.0	2.3

DM—dry matter.

**Table 3:** Data analysis according to transition periods.

Dependent Variable			Mean Difference	Std. Error	Sig. *p*
Rumination time	3-week period before calving	Calving day (±3 d)	31.77	20.21	0.12
		3-week period after calving	−68.84 *	14.29	<0.001
	3-week period after calving	3-week period before calving	68.84 *	14.29	<0.001
		Calving day (±3 d)	−100.62 *	25.14	<0.001
Eating time	3-week period before calving	Calving day (±3 d)	32.60	25.14	0.20
		3-week period after calving	−36.05 *	17.78	0.05
	3-week period after calving	3-week period before calving	36.05 *	17.78	0.05
		Calving day (±3 d)	68.64 *	25.14	0.01
Rumination chews	3-week period before calving	Calving day (±3 d)	2357.13	1494	0.12
		3-week period after calving	−4429.39 *	1056.42	<0.001
	3-week period after calving	3-week period before calving	4429.39 *	1056.42	<0.001
		Calving day (±3 d)	6786.51 *	1494	<0.001
Eating chews	3-week period before calving	Calving day (±3 d)	2431.69	2067.54	0.24
		3-week period after calving	−3325.05 *	1461.97	0.03
	3-week period after calving	3-week period before calving	3325.05 *	1461.97	0.03
		Calving day (±3 d)	5756.74 *	2067.54	0.01
Drinking gulps	3-week period before calving	Calving day (±3 d)	917.05	682.54	0.18
		3-week period after calving	−552.72	482.63	0.26
	3-week period after calving	3-week period before calving	552.72	482.63	0.26
		Calving day (±3 d)	1469.77 *	682.54	0.03
Bolus	3-week period before calving	Calving day (±3 d)	36.62	24.22	0.13
		3-week period after calving	−58.39 *	17.13	<0.001
	3-week period after calving	3-week period before calving	58.39 *	17.13	<0.001
		Calving day (±3 d)	95.00 *	24.22	<0.001
Chews per minute	3-week period before calving	Calving day (±3 d)	99.80	59.51	0.10
		3-week period after calving	−82.03	42.08	0.05
	3-week period after calving	3-week period before calving	82.03	42.08	0.05
		Calving day (±3 d)	181.83 *	59.51	0.01

* The mean difference is significant at the 0.05 level.

**Table 4:** Correlation between methane production (ppm) and Rumiwatch sensor indicators during three transition periods: three weeks before calving, calving day (±3 d), and three weeks after calving.

Correlations						
Methane Emission Across Periods						
Indices	3 Weeks Before Calving	Sig. *p*	Calving Day (±3 d)	Sig. *p*	3 Weeks After Calving	Sig. *p*
Eating time	0.12	0.478	−0.11	0.724	0.25	0.881
Drinking time	0.41 **	0.009	−0.35	0.239	0.04	0.824
Rumination chews	−0.09	0.603	0.37	0.210	−0.17	0.317
Eating chews	0.05	0.754	−0.16	0.592	0.01	0.938
Drinking gulps	0.01	0.983	−0.09	0.768	−0.27	0.091
Bolus	−0.07	0.608	0.43	0.148	−0.18	0.275
Chews per minute	−0.36 *	0.025	0.41	0.165	−0.08	0.652
Chews per bolus	0.31	0.058	−0.28	0.360	0.13	0.438
Activity	−0.24	0.139	−0.34	0.255	−0.22	0.173

* Correlation, r (Pearson’s correlation coefficient), is significant at *p* < 0.05 level (2-tailed). ** Correlation, r (Pearson’s correlation coefficient), is significant at *p* < 0.01 level (2-tailed). d—day.

**Table 5:** Correlation between methane production (ppm) and blood gas and additional metabolic parameters during three transition periods: three weeks before calving, calving day (±3 days), and three weeks after calving.

Correlations						
Methane Emission Across Periods						
Indices	3 Weeks Before Calving	Sig. *p*	Calving Day (±3 d)	Sig. *p*	3 Weeks After Calving	Sig. *p*
pH	0.01	0.976	0.23	0.450	0.07	0.672
pCO_2_	−0.04	0.831	−0.16	0.610	0.12	0.450
pO_2_	0.03	0.881	0.46	0.112	0.19	0.239
cHCO_3_^−^	0.17	0.311	0.05	0.870	−0.02	0.918
BE (ecf)	0.37 *	0.020	0.14	0.653	−0.05	0.769
cSO_2_	0.23	0.159	0.41	0.169	0.05	0.756
Na	−0.06	0.718	−0.15	0.614	0.09	0.583
K	0.2	0.224	0.29	0.330	0.1	0.556
Ca	−0.33 *	0.041	−0.22	0.466	−0.27	0.095
Cl	−0.42 **	0.008	−0.33	0.264	−0.13	0.428
TCO_2_	0.29	0.077	0.11	0.720	0.01	0.970
Hct	0.21	0.200	0.25	0.405	0.41 **	0.009
Hgb	−0.13	0.444	−0.51	0.075	−0.47 **	0.002
BE (b)	−0.1	0.535	−0.2	0.508	−0.36 *	0.027
Glu	−0.41 **	0.009	−0.24	0.431	0.2	0.231
Lac	0.12	0.469	0.1	0.756	−0.18	0.282
BUN	0.09	0.600	−0.13	0.668	0.03	0.871
Urea	0.2	0.218	−0.11	0.726	0.04	0.814
Crea	0.14	0.388	−0.35	0.240	−0.07	0.666

d—day; pH—hydrogen potential; pCO_2_—partial pressure of carbon dioxide; pO_2_—partial pressure of oxygen; cHCO_3_^−^—concentration of bicarbonate; BE (ecf)—base excess of extracellular fluid; cSO_2_—oxygen saturation of haemoglobin; Na—sodium; K—potassium; Ca—calcium; Cl—chlorides; tCO_2_—total carbon dioxide; Hct—haematocrit; Hgb—haemoglobin; BE (b) —base excess in blood; Glu—glucose; Lac—lactate; BUN—blood urea nitrogen; Urea—urea nitrogen; Crea—creatinine. * Correlation, r (Pearson’s correlation coefficient), is significant at *p* < 0.05 level (2-tailed). ** Correlation, r (Pearson’s correlation coefficient), is significant at *p* < 0.01 level (2-tailed).

**Table 6:** Correlation between methane production (ppm) and blood morphological and haematological parameters during three transition periods: three weeks before calving, calving day (±3 days), and three weeks after calving.

Correlations						
Methane Emission Across Periods						
Indices	3 Weeks Before Calving	Sig. *p*	Calving Day (±3 d)	Sig. *p*	3 Weeks After Calving	Sig. *p*
WBC	0.21	0.194	−0.37	0.220	−0.06	0.736
LYM	−0.03	0.846	−0.08	0.805	−0.14	0.408
MON	0.50 **	0.001	−0.41	0.164	0.2	0.215
Neu	0.3	0.067	−0.32	0.285	−0.06	0.704
Eos	−0.21	0.207	−0.16	0.614	−0.17	0.298
Bas	0.09	0.567	−0.31	0.298	−0.28	0.082
LYM%	−0.01	0.958	0.27	0.382	−0.05	0.764
MON%	0.46 **	0.004	−0.36	0.223	0.22	0.186
Neu%	0.16	0.333	−0.16	0.597	0.04	0.806
Eos%	−0.2	0.234	0.04	0.892	−0.12	0.484
Bas%	0.05	0.784	−0.35	0.247	−0.25	0.132
Rbc	−0.19	0.257	−0.14	0.646	−0.12	0.479
Hgb	−0.25	0.126	0.27	0.367	−0.01	0.935
Hct	−0.02	0.890	0.43	0.140	−0.01	0.995
Mcv	0.21	0.203	0.49	0.087	0.11	0.511
MCH	−0.01	0.934	0.36	0.233	0.16	0.326
Mchc	−0.23	0.160	−0.4	0.182	−0.02	0.930
PLT	0.26	0.118	−0.64 *	0.018	−0.41 **	0.009

* Correlation, r (Pearson’s correlation coefficient), is significant at *p* < 0.05 level (2-tailed). ** Correlation, r (Pearson’s correlation coefficient), is significant at *p* < 0.01 level (2-tailed). d—day; WBC—white blood cells, ×10⁹/L; LYM—lymphocytes, ×10⁹/L; MON—monocytes, ×10⁹/L; Neu—neutrophils, ×10^9^/L; Eos—eosinophils, ×10⁹/L; Bas—basophils, ×10^9^/L; LYM%—percentage of lymphocytes, %; MON%—percentage of monocytes, %; Neu%—percentage of neutrophils, %; Eos%—percentage of eosinophils, %; Bas%—percentage of basophils, %; Rbc—red blood cells, ×10^12^/L; Hgb—haemoglobin, g/L; Hct—haematocrit, %; Mcv—mean corpuscular volume, fL; MCH—mean corpuscular haemoglobin, pg; MCHC—mean corpuscular haemoglobin concentration, g/L; PLT—platelets, ×10^9^/L.

**Table 7:** Correlation between methane production (ppm) and Rumiwatch sensor indicators in cows with low (<404 min/d), medium (404–500 min/d), and high (>500 min/d) rumination durations.

Correlations						
Methane Emissions in Groups with Different Rumination Times						
Indices	<404 min/d	Sig. *p*	404–500 min/d	Sig. *p*	>500 min/d	Sig. *p*
Eating time	0.26	0.347	0.02	0.912	−0.05	0.772
Drinking time	0.15	0.587	0.01	0.985	0.18	0.347
Rumination chews	−0.52 *	0.046	−0.17	0.256	0.04	0.818
Eating chews	0.18	0.512	−0.02	0.895	−0.07	0.716
Drinking gulps	−0.22	0.438	−0.02	0.991	−0.42 *	0.019
Bolus	0.25	0.373	0.16	0.307	−0.37 *	0.043
Chews per minute	−0.10	0.721	−0.08	0.583	−0.17	0.362
Chews per bolus	−0.03	0.925	0.03	0.840	0.28	0.125
Activity	−0.36	0.188	−0.25	0.103	−0.31	0.086

* Correlation, r (Pearson’s correlation coefficient), is significant at *p* < 0.05 level (2-tailed).

**Table 8:** Principal Component Analysis on Rumiwatch noseband sensor indicators.

Component	Initial Eigenvalues			Extraction Sums of Squared Loadings			Rotation Sums of Squared Loadings ^a^	
	Total	% of Variance	Cumulative %	Total	% of Variance	Cumulative %	Total	
1	5.34	28.10	28.10	5.34	28.10	28.10	4.49	
2	2.95	15.52	43.62	2.95	15.52	43.62	3.96	
3	2.36	12.39	56.01	2.36	12.39	56.01	2.41	
4	1.91	10.05	66.06	1.91	10.10	66.06	2.01	
5	1.59	8.34	74.40	1.59	8.34	74.40	1.91	
6	1.06	5.57	79.97	1.06	5.57	79.97	1.69	
7	0.91	4.76	84.74					
8	0.7	3.69	88.42					
9	0.59	3.09	91.51					
10	0.47	2.48	93.99					
11	0.43	2.26	96.25					
12	0.26	1.39	97.63					
13	0.17	0.90	98.54					
14	0.1	0.53	99.06					
15	0.09	0.47	99.54					
16	0.05	0.28	99.82					
17	0.02	0.11	99.93					
18	0.01	0.04	99.97					
19	0.01	0.03	100					

Extraction method: Principal Component Analysis. a—when components are correlated, sums of squared loadings cannot be added to obtain total variance.

**Table 9:** Pattern matrix generated through PCA using Oblimin rotation, exhibiting distinctive relationships between various cow behavioural metrics and methane emissions.

Pattern Matrix ^a^						
	Component					
	1	2	3	4	5	6
Bolus	0.92	−0.03	−0.02	−0.07	−0.11	−0.05
Rumination time	0.91	−0.11	−0.04	0.04	−0.17	0.06
Rumination chew	0.91	−0.14	0.03	0.07	−0.02	0.03
Chews per minute	0.90	0.20	0.02	0.07	0.18	0.01
Down time	0.59	0.06	−0.01	−0.25	0.09	0.11
Eating chews 1	−0.12	−0.96	−0.07	−0.05	−0.06	0.13
Eating time 1	−0.14	−0.96	−0.06	−0.00	−0.11	0.15
Drinking gulp	0.20	−0.75	−0.08	0.03	0.26	−0.16
Other activity time	−0.44	0.67	−0.18	−0.01	0.20	−0.13
Up time	0.13	−0.58	0.24	0.11	0.25	−0.04
Eating time 2	−0.05	0.03	0.98	−0.07	−0.06	0.05
Eating chews 2	0.03	−0.03	0.97	−0.06	−0.03	−0.02
Chews per bolus	−0.08	0.04	−0.04	0.95	−0.01	0.05
Drinking time	0.04	−0.07	−0.07	0.94	−0.08	−0.01
Activity	−0.01	−0.11	0.25	−0.10	0.78	0.04
Other chew	0.08	0.51	0.02	0.13	0.65	0.06
Methane	0.06	0.11	0.28	0.11	−0.61	0.03
Activity change	−0.06	0.00	0.26	0.09	0.32	0.77

Extraction method: Principal Component Analysis. Rotation method: Oblimin with Kaiser normalisation. a—rotation converged in 10 iterations.

**Table 10:** DMI, methane emissions, and methane yields across transition periods in dairy cows.

Transition Period	Mean DMI (kg/day)	Mean Methane (ppm)	Methane Yield (ppmCH_4_/kg DMI)
Prepartum	13.92 ± 2.87	391.38 ± 13.43	28.11 ± 3.2 ^b^
Calving Day	6.12 ± 1.83	422.27 ± 44.11	69.01 ± 5.6 ^a^
Postpartum	21.34 ± 3.52	408.68 ± 15.36	19.15 ± 2.8 ^b^

^a,b^ indicate statistically significant differences (*p* < 0.001) in methane yield across periods. DMI—dry matter intake; ppmCH_4_/kg DMI—methane yield (parts per million of methane per kilogram of dry matter intake).

**Table 11:** Correlations between DMI, methane emission, and rumination time in dairy cows.

Indices	Methane Emission (ppm)	Methane Yield (ppmCH_4_/kg DMI)
DMI	0.35	−0.42 *

* Correlation, r (Pearson’s correlation coefficient), is significant at *p* < 0.05 level (2-tailed). DMI—dry matter intake; ppmCH_4_/kg DMI—methane yield (parts per million of methane per kilogram of dry matter intake).

## Data Availability

The data provided in this study can be found in the publication.

## References

[1] The European Green Deal. https://ec.europa.eu/commission/presscorner/detail/en/ip_19_6691.

[2] Bačėninaitė D., Džermeikaitė K., Antanaitis R. (2022). Global Warming and Dairy Cattle: How to Control and Reduce Methane Emission. Animals.

[3] US EPA Agriculture and Aquaculture: Food for Thought. https://www.epa.gov/snep/agriculture-and-aquaculture-food-thought.

[4] Can We Make Cow Burps Climate-Friendly?|Horizon Magazine. https://projects.research-and-innovation.ec.europa.eu/en/horizon-magazine/can-we-make-cow-burps-climate-friendly.

[5] Lassen J., Løvendahl P. (2016). Heritability Estimates for Enteric Methane Emissions from Holstein Cattle Measured Using Noninvasive Methods. J. Dairy Sci..

[6] Aryee G., Luecke S.M., Dahlen C.R., Swanson K.C., Amat S. (2023). Holistic View and Novel Perspective on Ruminal and Extra-Gastrointestinal Methanogens in Cattle. Microorganisms.

[7] Watt L.J., Clark C.E.F., Krebs G.L., Petzel C.E., Nielsen S., Utsumi S.A. (2015). Differential Rumination, Intake, and Enteric Methane Production of Dairy Cows in a Pasture-Based Automatic Milking System. J. Dairy Sci..

[8] Zetouni L., Difford G.F., Lassen J., Byskov M.V., Norberg E., Løvendahl P. (2018). Is Rumination Time an Indicator of Methane Production in Dairy Cows?. J. Dairy Sci..

[9] O’Connor S., Noonan F., Savage D., Walsh J. (2024). Advancements in Real-Time Monitoring of Enteric Methane Emissions from Ruminants. Agriculture.

[10] Tsiamadis V., Kougioumtzis A., Siachos N., Panousis N., Kritsepi-Konstantinou M., Valergakis G.E. (2022). Hematology Reference Intervals during the Prepartum Period, First Week after Calving, and Peak Lactation in Clinically Healthy Holstein Cows. Vet. Clin. Pathol..

[11] Sorg D. (2022). Measuring Livestock CH4 Emissions with the Laser Methane Detector: A Review. Methane.

[12] Mikuła R., Pszczola M., Rzewuska K., Mucha S., Nowak W., Strabel T. (2022). The Effect of Rumination Time on Milk Performance and Methane Emission of Dairy Cows Fed Partial Mixed Ration Based on Maize Silage. Animals.

[13] Lopes L.S.F., Schenkel F.S., Houlahan K., Rochus C.M., Oliveira G.A., Oliveira H.R., Miglior F., Alcantara L.M., Tulpan D., Baes C.F. (2024). Estimates of Genetic Parameters for Rumination Time, Feed Efficiency, and Methane Production Traits in First-Lactation Holstein Cows. J. Dairy Sci..

[14] Danielsson R., Dicksved J., Sun L., Gonda H., Müller B., Schnürer A., Bertilsson J. (2017). Methane Production in Dairy Cows Correlates with Rumen Methanogenic and Bacterial Community Structure. Front. Microbiol..

[15] Olijhoek D.W., Løvendahl P., Lassen J., Hellwing A.L.F., Höglund J.K., Weisbjerg M.R., Noel S.J., McLean F., Højberg O., Lund P. (2018). Methane Production, Rumen Fermentation, and Diet Digestibility of Holstein and Jersey Dairy Cows Being Divergent in Residual Feed Intake and Fed at 2 Forage-to-Concentrate Ratios. J. Dairy Sci..

[16] Arfuso F., Minuti A., Liotta L., Giannetto C., Trevisi E., Piccione G., Lopreiato V. (2023). Stress and Inflammatory Response of Cows and Their Calves during Peripartum and Early Neonatal Period. Theriogenology.

[17] Merdana I.M., Sulabda I., Tiasnitha N., Gunawan N., Sudira I. (2020). Erythrocyte, Hemoglobin and Hematocrit Profile of Bali Cattle during the Various Periods of Parturition. J. Anim. Health Prod..

[18] Pascottini O.B., Leroy J.L.M.R., Opsomer G. (2020). Metabolic Stress in the Transition Period of Dairy Cows: Focusing on the Prepartum Period. Animals.

[19] Morgavi D.P., Cantalapiedra-Hijar G., Eugène M., Martin C., Noziere P., Popova M., Ortigues-Marty I., Muñoz-Tamayo R., Ungerfeld E.M. (2023). Review: Reducing Enteric Methane Emissions Improves Energy Metabolism in Livestock: Is the Tenet Right?. Animal.

[20] Meese S., Ulbrich S.E., Bollwein H., Bruckmaier R., Wellnitz O., Kreuzer M., Röntgen M., Gimsa U., Schwarm A. (2020). Methane Emission, Metabolism, and Performance of Holstein Dairy Cows with Low, Medium, and High Lymphocyte Proliferation during Transition. J. Dairy Sci..

[21] RO Y., CHOI W., PARK J., CHOE E., KIM D. (2020). Changes in Plasma pH and Blood and Urinary Macromineral Concentrations in Experimentally Induced Hypocalcemic Cows with Na2EDTA. J. Vet. Med. Sci..

[22] Goff J.P. (2008). The Monitoring, Prevention, and Treatment of Milk Fever and Subclinical Hypocalcemia in Dairy Cows. Vet. J..

[23] National Research Council (2001). Nutrient Requirements of Dairy Cattle: Seventh Revised Edition.

[24] Hu W., Murphy M.R. (2004). Dietary Cation-Anion Difference Effects on Performance and Acid-Base Status of Lactating Dairy Cows: A Meta-Analysis. J. Dairy Sci..

[25] Sorg D., Mühlbach S., Rosner F., Kuhla B., Derno M., Meese S., Schwarm A., Kreuzer M., Swalve H. (2017). The Agreement between Two Next-Generation Laser Methane Detectors and Respiration Chamber Facilities in Recording Methane Concentrations in the Spent Air Produced by Dairy Cows. Comput. Electron. Agric..

[26] A Portable Remote Methane Detector Using an InGaAsP DFB Laser|Environmental Geology. https://link.springer.com/article/10.1007/s00254-004-1094-0.

[27] Bao J., Wang L., Li S., Guo J., Ma P., Huang X., Guo G., Zhang H., Wang Y. (2024). Screening and Functional Prediction of Rumen Microbiota Associated with Methane Emissions in Dairy Cows. Animals.

[28] Antanaitis R., Anskienė L., Rapaliutė E., Bilskis R., Džermeikaitė K., Bačėninaitė D., Juškienė V., Juška R., Meškinytė E. (2022). Relationship between Reticulorumen Parameters Measured in Real Time and Methane Emission and Heat Stress Risk in Dairy Cows. Animals.

[29] Hesai Technology PandarQT User Manual Q01.

[30] Fadul M., D’Andrea L., Alsaaod M., Borriello G., Di Lori A., Stucki D., Ciaramella P., Steiner A., Guccione J. (2022). Assessment of Feeding, Ruminating and Locomotion Behaviors in Dairy Cows around Calving—A Retrospective Clinical Study to Early Detect Spontaneous Disease Appearance. PLoS ONE.

[31] Antanaitis R., Anskienė L., Palubinskas G., Džermeikaitė K., Bačėninaitė D., Viora L., Rutkauskas A. (2023). Ruminating, Eating, and Locomotion Behavior Registered by Innovative Technologies around Calving in Dairy Cows. Animals.

[32] Zehner N., Umstätter C., Niederhauser J.J., Schick M. (2017). System Specification and Validation of a Noseband Pressure Sensor for Measurement of Ruminating and Eating Behavior in Stable-Fed Cows. Comput. Electron. Agric..

[33] Antanaitis R., Džermeikaitė K., Bespalovaitė A., Ribelytė I., Rutkauskas A., Japertas S., Baumgartner W. (2023). Assessment of Ruminating, Eating, and Locomotion Behavior during Heat Stress in Dairy Cattle by Using Advanced Technological Monitoring. Animals.

[34] Proudfoot K.L., Huzzey J.M. (2022). A First Time for Everything: The Influence of Parity on the Behavior of Transition Dairy Cows*. JDS Commun..

[35] Hendriks S.J., Phyn C.V.C., Turner S.-A., Mueller K.M., Kuhn-Sherlock B., Donaghy D.J., Huzzey J.M., Roche J.R. (2019). Lying Behavior and Activity during the Transition Period of Clinically Healthy Grazing Dairy Cows. J. Dairy Sci..

[36] Campler M.R., Munksgaard L., Jensen M.B. (2019). The Effect of Transition Cow Housing on Lying and Feeding Behavior in Holstein Dairy Cows. J. Dairy Sci..

[37] Miller G.A., Mitchell M., Barker Z.E., Giebel K., Codling E.A., Amory J.R., Michie C., Davison C., Tachtatzis C., Andonovic I. (2020). Using Animal-Mounted Sensor Technology and Machine Learning to Predict Time-to-Calving in Beef and Dairy Cows. Animal.

[38] Giaretta E., Marliani G., Postiglione G., Magazzù G., Pantò F., Mari G., Formigoni A., Accorsi P.A., Mordenti A. (2021). Calving Time Identified by the Automatic Detection of Tail Movements and Rumination Time, and Observation of Cow Behavioural Changes. Animal.

[39] Rombach M., Münger A., Niederhauser J., Südekum K.-H., Schori F. (2018). Evaluation and Validation of an Automatic Jaw Movement Recorder (RumiWatch) for Ingestive and Rumination Behaviors of Dairy Cows during Grazing and Supplementation. J. Dairy Sci..

[40] Li Z., Cheng L., Cullen B. (2021). Validation and Use of the RumiWatch Noseband Sensor for Monitoring Grazing Behaviours of Lactating Dairy Cows. Dairy.

[41] Macmillan K., Gobikrushanth M., Colazo M.G. (2022). Activity and Rumination Changes as Predictors of Calving in Primiparous and Multiparous Holstein Cows. Livest. Sci..

[42] Soriani N., Trevisi E., Calamari L. (2012). Relationships between Rumination Time, Metabolic Conditions, and Health Status in Dairy Cows during the Transition Period1. J. Anim. Sci..

[43] Knapp J.R., Laur G.L., Vadas P.A., Weiss W.P., Tricarico J.M. (2014). *Invited Review:* Enteric Methane in Dairy Cattle Production: Quantifying the Opportunities and Impact of Reducing Emissions. J. Dairy Sci..

[44] Aschenbach J.R., Kristensen N.B., Donkin S.S., Hammon H.M., Penner G.B. (2010). Gluconeogenesis in Dairy Cows: The Secret of Making Sweet Milk from Sour Dough. IUBMB Life.

[45] Kim M., Masaki T., Ikuta K., Iwamoto E., Nishihara K., Hirai M., Uemoto Y., Terada F., Roh S. (2022). Physiological Responses and Adaptations to High Methane Production in Japanese Black Cattle. Sci. Rep..

[46] Hristov A.N., Oh J., Firkins J.L., Dijkstra J., Kebreab E., Waghorn G., Makkar H.P.S., Adesogan A.T., Yang W., Lee C. (2013). SPECIAL TOPICS—Mitigation of Methane and Nitrous Oxide Emissions from Animal Operations: I. A Review of Enteric Methane Mitigation Options1. J. Anim. Sci..

[47] Reintke J., Brügemann K., Yin T., Wagner H., Wehrend A., Müller A., König S. (2021). Associations between Minerals and Metabolic Indicators in Maternal Blood Pre- and Postpartum with Ewe Body Condition, Methane Emissions, and Lamb Body Weight Development. Animal.

[48] Gul F., Amin H., Naz S., Khan M.T., Alhidary I.A., Khan R.U., Pugliese G., Tufarelli V. (2024). Evaluation of Blood Minerals and Oxidative Stress Changing Pattern in Prepartum and Postpartum Achai and Holstein Friesian Dairy Cows. Reprod. Domest. Anim..

[49] Zurr L., Leonhard-Marek S. (2012). Effects of β-Hydroxybutyrate and Different Calcium and Potassium Concentrations on the Membrane Potential and Motility of Abomasal Smooth Muscle Cells in Cattle. J. Dairy Sci..

[50] Hansen S.S., Nørgaard P., Pedersen C., Jørgensen R.J., Mellau L.S.B., Enemark J.D. (2003). The Effect of Subclinical Hypocalcaemia Induced by Na2EDTA on the Feed Intake and Chewing Activity of Dairy Cows. Vet. Res. Commun..

[51] Yin G., Sun Z., Wang Z., Xia Y., Cheng L., Qin G., Aschalew N.D., Liu H., Zhang X., Wu Q. (2024). Mechanistic Insights into Inositol-Mediated Rumen Function Promotion and Metabolic Alteration Using in Vitro and in Vivo Models. Front. Vet. Sci..

[52] White H.M., Carvalho E.R., Koser S.L., Schmelz-Roberts N.S., Pezzanite L.M., Slabaugh A.C., Doane P.H., Donkin S.S. (2016). Short Communication: Regulation of Hepatic Gluconeogenic Enzymes by Dietary Glycerol in Transition Dairy Cows. J. Dairy Sci..

[53] Joo S.S., Lee S.J., Park D.S., Kim D.H., Gu B.-H., Park Y.J., Rim C.Y., Kim M., Kim E.T. (2021). Changes in Blood Metabolites and Immune Cells in Holstein and Jersey Dairy Cows by Heat Stress. Animals.

[54] Cai J., Wang D., Liu J. (2018). Regulation of Fluid Flow through the Mammary Gland of Dairy Cows and Its Effect on Milk Production: A Systematic Review. J. Sci. Food Agric..

[55] Moretti P., Paltrinieri S., Trevisi E., Probo M., Ferrari A., Minuti A., Giordano A. (2017). Reference Intervals for Hematological and Biochemical Parameters, Acute Phase Proteins and Markers of Oxidation in Holstein Dairy Cows around 3 and 30 Days after Calving. Res. Vet. Sci..

[56] Castaneda A., Indugu N., Lenker K., Narayan K., Rassler S., Bender J., Baker L., Purandare O., Chai D., Webb T. (2025). Investigating Rumination and Eating Time as Proxies for Identifying Dairy Cows with Low Methane Emitting Potential. JDS Commun..

[57] López-Paredes J., Goiri I., Atxaerandio R., García-Rodríguez A., Ugarte E., Jiménez-Montero J.A., Alenda R., González-Recio O. (2020). Mitigation of Greenhouse Gases in Dairy Cattle via Genetic Selection: 1. Genetic Parameters of Direct Methane Using Noninvasive Methods and Proxies of Methane. J. Dairy Sci..

[58] Kaufman E.I., Asselstine V.H., LeBlanc S.J., Duffield T.F., DeVries T.J. (2018). Association of Rumination Time and Health Status with Milk Yield and Composition in Early-Lactation Dairy Cows. J. Dairy Sci..

[59] Beauchemin K.A. (2018). *Invited Review:* Current Perspectives on Eating and Rumination Activity in Dairy Cows. J. Dairy Sci..

[60] Byskov M.V., Nadeau E., Johansson B.E.O., Nørgaard P. (2015). Variations in Automatically Recorded Rumination Time as Explained by Variations in Intake of Dietary Fractions and Milk Production, and between-Cow Variation. J. Dairy Sci..

[61] Cameron L., Chagunda M.G.G., Roberts D.J., Lee M.A. (2018). A Comparison of Milk Yields and Methane Production from Three Contrasting High-Yielding Dairy Cattle Feeding Regimes: Cut-and-Carry, Partial Grazing and Total Mixed Ration. Grass Forage Sci..

[62] Cantor M.C., Costa J.H.C., Bewley J.M. (2018). Impact of Observed and Controlled Water Intake on Reticulorumen Temperature in Lactating Dairy Cattle. Animals.

[63] Brod D.L., Bolsen K.K., Brent B.E. (1982). Effect of Water Temperature in Rumen Temperature, Digestion and Rumen Fermentation in Sheep. J. Anim. Sci..

[64] Grossi S., Rossi L., Dell’Anno M., Biffani S., Sgoifo Rossi C.A. (2021). Effects of Heated Drinking Water on the Growth Performance and Rumen Functionality of Fattening Charolaise Beef Cattle in Winter. Animals.

